# Regional anesthesia in colorectal laparoscopy: a retrospective comparison of quadratus lumborum and TAP blocks

**DOI:** 10.25122/jml-2025-0067

**Published:** 2025-04

**Authors:** Mihaela Roxana Oliță, Mihai Adrian Eftimie, Andrei Andreșanu, Mihai Adrian Dobra, Elena Liliana Mirea, Dana Rodica Tomescu

**Affiliations:** 1 Anesthesiology and Intensive Care, Fundeni Clinical Institute, Bucharest, Romania; 2 Carol Davila University of Medicine and Pharmacy, Bucharest, Romania; 3 Department of Surgery, Fundeni Clinical Institute, Bucharest, Romania; 4 Department of Urology, Fundeni Clinical Institute, Bucharest, Romania; 5 Anesthesiology and Intensive Care Clinic, Clinical Emergency Hospital Bucharest, Bucharest, Romania

**Keywords:** quadratus lumborum block, transversus abdominis plane block, laparoscopic colorectal surgery, postoperative analgesia, regional anesthesia, pain management, retrospective study, EOM, External Oblique Muscle, IOM, Internal Oblique Muscle, LA, Local Anesthetic, PACU, Post Anesthesia Care Unit, QLM, Quadratus Lumborum Muscle, TAM, Transversus Abdominis Muscle, TAP, Transversus Abdominis Plane, VAS, Visual Analogue Scale, ESM, Erector Spinae Muscle, PMM, Psoas Major Muscle, US, Ultrasound, EBL, Estimated Blood Loss, LOS, Length Of Stay

## Abstract

Effective postoperative pain control is pivotal in enhancing recovery following laparoscopic colorectal surgery. Regional anesthesia techniques such as the transversus abdominis plane (TAP) block and the quadratus lumborum (QL) block have gained prominence as components of multimodal analgesia. However, their comparative efficacy remains underexplored. This retrospective observational cohort study analyzed data from 289 patients undergoing laparoscopic colon surgery. Patients were stratified into three groups: TAP block (Group A, *n* = 54), QL block (Group B, *n* = 62), and no regional block (Group C, *n* = 173). Primary endpoints included time to first analgesic administration and total analgesic consumption (opioids and non-opioids). Statistical analyses were conducted using R (v4.4.2) and Jamovi (v2.3), with significance set at *P* < 0.05. Group B (QL block) demonstrated significantly reduced opioid consumption (mean 13.16 ± 2.69 mg) compared to both Group A (16.80 ± 5.51 mg) and Group C (18.03 ± 4.29 mg), *P* < 0.001. Time to first analgesic request was longer in Group B (16.06 ± 2.53 h), indicating more durable analgesia. Non-opioid usage (paracetamol, tramadol, nefopam) was similarly lower in Group B across all comparisons (*P* < 0.001). Group B also exhibited a significantly shorter hospital stay (4.87 ± 1.14 days) relative to Groups A and C. The QL block was associated with superior postoperative analgesia, reduced opioid and adjunct analgesic requirements, prolonged pain-free intervals, and accelerated postoperative recovery in laparoscopic colorectal surgery. These findings underscore QL block as a potent element of opioid-sparing, multimodal analgesic strategies and support its broader adoption in enhanced recovery protocols.

## INTRODUCTION

Effective postoperative analgesia remains a cornerstone of enhanced recovery protocols in abdominal surgery, particularly within the domain of colorectal procedures. In recent decades, the evolution of minimally invasive surgical techniques, such as laparoscopy, has markedly reduced operative trauma and shortened hospital stays [1]. Nevertheless, even with laparoscopic approaches, patients frequently report moderate to severe postoperative pain due to a combination of incisional, visceral, and referred discomfort [2]. In the context of laparoscopic colorectal surgery, nociceptive stimuli arise from trocar insertion sites, peritoneal insufflation, mesenteric traction, and bowel manipulation, necessitating a carefully balanced multimodal analgesic regimen [3,4].

Traditionally, systemic opioids have been the mainstay of postoperative pain control. However, opioid-related adverse effects—such as ileus, sedation, nausea, vomiting, urinary retention, and risk of prolonged dependence—have prompted the search for alternative strategies [5,6]. Within this framework, regional anesthesia techniques have gained prominence for their ability to provide site-specific analgesia while reducing systemic drug exposure. Among these, the transversus abdominis plane (TAP block has been widely adopted due to its ease of administration and efficacy in controlling somatic pain originating from the anterior abdominal wall [7-9].

Despite its benefits, the TAP block’s analgesic coverage is largely limited to the T10–L1 dermatomes and offers minimal visceral pain relief. Consequently, attention has shifted toward more versatile fascial plane blocks capable of broader and deeper anesthetic spread [10-12]. The QL block, first described in 2007 and further refined into subtypes (lateral, posterior, anterior), has shown promise. The anterior QL block (Type 3) allows local anesthetic deposition between the quadratus lumborum and psoas major muscles, potentially facilitating diffusion toward the thoracolumbar fascia and paravertebral space [13,14]. This anatomical advantage translates into more comprehensive analgesia, potentially encompassing somatic and visceral components [15].

In recent years, many studies have highlighted the analgesic benefits of the quadratus lumborum (QL) block in diverse surgical contexts, including cesarean sections, nephrectomies, and ventral hernia repairs. Despite these advancements, there is a notable lack of focused literature on its application in laparoscopic oncologic colorectal surgery, with existing studies often exhibiting methodological inconsistencies, underscoring the need for further investigation into the efficacy of the QL block in specific surgical domains [16,17].

The study aimed to compare the analgesic efficacy associated with anterior QL block versus TAP block in patients undergoing elective laparoscopic oncologic colorectal resections, specifically focusing on time to first analgesic, opioid-sparing effect, and hospitalization duration.

## MATERIAL AND METHODS

### Study design and ethical considerations

This single-center retrospective study was conducted at the Department of Oncologic and Hepatobiliopancreatic Surgery, Fundeni Clinical Institute, Bucharest, Romania, one of the leading tertiary surgical centers in Eastern Europe. Ethical approval was obtained from the institutional ethics committee (Study No. 1891/2025), and all procedures adhered to the principles outlined in the Declaration of Helsinki. Patient data were collected exclusively from existing clinical records, ensuring confidentiality and compliance with institutional data governance protocols. Data extraction was performed by members of the anesthesiology department, primarily residents operating under direct senior supervision, thereby minimizing inter-observer variability and enhancing data integrity.

### Patient selection criteria

Between January 2020 and December 2024, a total of 289 adult patients (aged 18–90 years) underwent elective laparoscopic oncologic colorectal resections under general anesthesia at our institution. Peripheral nerve blocks are routinely employed as part of multimodal analgesia, with block selection determined by the attending anesthesiologist within a consistent institutional framework. The final analytical sample was stratified into three groups based on the perioperative analgesic modality:

- *Group A* (*n* = 54): TAP block
- *Group B* (*n* = 62): QL block
- *Group C* (*n* = 173): No regional block

### Inclusion criteria

Complete clinical documentation, elective surgery indication, and standardized perioperative management. Patients received peripheral nerve blocks after surgical closure and before extubation, following departmental protocol.

### Exclusion criteria

- Emergency surgeries (e.g., obstruction, perforation)
- Conversion to open surgery
- Metastatic disease or synchronous colorectal tumors
- Additional malignancies or major prior abdominal surgeries
- Severe anemia (Hb < 7 g/dL) or intraoperative hemodynamic instability
- Unplanned intraoperative thoracic epidural placement

### Anesthetic and surgical technique

All data regarding this section can be found in Supplementary Data (S1).

S1 - Anesthetic and Surgical Technique

### Regional anesthesia techniques

#### TAP block (Group A, *n* = 54)

Bilateral TAP blocks were performed using a linear probe positioned at the midaxillary line between the iliac crest and costal margin. The needle was advanced in-plane into the fascial plane between the internal oblique muscle (IOM) and the transversus abdominis muscle (TAM). After confirming the correct needle placement, 20 mL of 0.25% ropivacaine was administered on each side. An effective block was verified by the hypoechoic spread between the IOM and TAM.

#### QL block (Group B, *n* = 62)

Bilateral anterior QL (Type 3) blocks were performed under ultrasound (US) guidance using a low-frequency curved transducer. With the patient in the lateral decubitus position, the 'shamrock sign' — comprising the quadratus lumborum muscle (QLM), psoas major muscle (PMM), and erector spinae muscle (ESM)—was visualized. Then, 20 mL of 0.25% bupivacaine was deposited between the anterior fascia of the QLM and the PMM at the L2–L3 vertebral level. A successful block was defined by a clear sonographic spread of the injectate bilaterally within the targeted plane.

Postoperative pain management was systematically evaluated by analyzing pain scores measured using the visual analog scale (VAS). Pain assessments were conducted at specific intervals (1, 2, 4, 8, 12, and 48 hours post-regional block administration), and evaluations were performed in two physiological states, at rest and during coughing, to capture static and dynamic pain components. Initial pain scores were documented one hour post-extubation, with subsequent assessments every 2 hours for the first 12 hours and every 4 hours thereafter until 48 hours postoperatively.

The duration of analgesia was defined as the interval from regional anesthetic administration to the first patient-initiated request for supplemental analgesia, and rescue analgesia was administered stepwise: intravenous paracetamol was the first-line agent for a VAS score ≥3. If pain persisted after 2 hours, intravenous diclofenac (1.5 mg/kg, max 150 mg/24 hours) was administered. For inadequate pain control, intravenous nefopam (max 60 mg/day) was subsequently provided, and for breakthrough pain unresponsive to these measures, intravenous morphine (0.1 mg/kg) was given based on clinical discretion. Data on opioid consumption within the first 48 hours, total non-opioid analgesics utilized, and time to first rescue analgesia were meticulously recorded.

### Data collection and documentation protocol

Clinical data were systematically extracted from standardized perioperative observation charts and electronic medical records. These forms, completed by anesthesiology residents, ensured consistent documentation of anesthetic, surgical, and postoperative analgesic variables. Data points included demographics, intraoperative parameters, and analgesic use (opioid and non-opioid).

### Statistical analysis

All statistical analyses were conducted using R statistical software, version 4.4.2 (The R Foundation for Statistical Computing, Vienna, Austria) and Jamovi software, version 2.3 (The jamovi project), with a predefined significance threshold of *P* < 0.05. Data integrity was verified through initial completeness checks and exploratory analyses to identify outliers and assess distributional assumptions.

Descriptive statistics were computed for all relevant demographic, intraoperative, and postoperative variables. Continuous variables were summarized using means and standard deviations (SD) or medians and interquartile ranges (IQR), depending on data normality, which was evaluated via the Shapiro–Wilk test and visual inspection of Q–Q plots. Categorical variables were presented as absolute frequencies and percentages. To compare continuous variables across the three study groups, one-way analysis of variance (ANOVA) was applied for normally distributed variables, followed by Games–Howell post-hoc analysis to account for unequal variances and sample sizes. In cases of non-normality, the Kruskal–Wallis H test was employed as a non-parametric alternative, supplemented by pairwise Wilcoxon rank-sum tests with Bonferroni correction for multiple comparisons.

Categorical variables (e.g., dose stratification of tramadol and Diclofenacum + Orphenandrinum citras) were analyzed using the Pearson chi-square test of independence. When expected cell counts were below 5, Fisher’s exact test was utilized to ensure statistical robustness.

To evaluate the effect of nerve block type on postoperative pain scores, which were measured at six distinct postoperative time points for each patient, a repeated-measures design was implemented. A Linear Mixed Effects Model (LMM) was considered optimal to account for intra-subject correlation and unbalanced data structure. Fixed effects included group assignment and time, while patient ID was treated as a random effect. Model fit was assessed using the Akaike Information Criterion (AIC) and residual diagnostics.

Analyses pertaining to time to first analgesic administration and total analgesic consumption were conducted using general linear models (GLMs), adjusted for potential confounders, including age, sex, surgical duration, and intraoperative blood loss, when appropriate. Although the QL block was administered postoperatively and is thus unlikely to exert direct intraoperative hemodynamic effects, this discrepancy may reflect institutional trends or subtle perioperative advantages associated with the cohort receiving QL blocks. Potential contributing factors include improved intraoperative stability due to anticipatory block planning or differential anesthetic agent dosing and fluid management influenced by anticipated block efficacy.

## RESULTS

This retrospective study highlights the significant impact of regional anesthesia techniques, particularly the QL block, on postoperative analgesia and recovery in patients undergoing laparoscopic colorectal surgery. The QL block was associated with reduced opioid and analgesic consumption, delayed time to first analgesic requirement, and shorter hospital stays compared to both TAP block and standard care. These findings suggest that the QL block may be an effective multimodal analgesia component in minimally invasive colorectal surgery (Table 1).

**Table 1:** Comparative table of clinical variables among anesthesia groups

Variable	Group A (TAP)	Group B (QL)	Group C (No block)	*P* value
**Age (years)**	68.09 ± 9.47	67.69 ± 7.92	66.64 ± 7.37	0. 452
**Surgery duration (min)**	244.44 ± 58.01	234.03 ± 55.14	248.61 ± 52.01	0.199
**Total opioid dose (mg)**	16.80 ± 5.51	13.16 ± 2.69	18.03 ± 4.29	<0.001
**Time to first analgesic (h)**	8.11 ± 1.09	16.06 ± 2.53	5.50 ± 1.42	<0.001
**Estimated blood loss (ml)**	272.59 ± 296.54	106.29 ± 69.99	239.31 ± 84.07	< 0.001
**Length of stay (days)**	6.91 ± 2.40	4.87 ± 1.14	5.46 ± 1.61	<0.001

No statistically significant differences were observed in mean patient age across the groups (*P* = 0.452), suggesting a comparable distribution of chronological age, a variable often associated with pain perception, pharmacokinetics, and recovery kinetics. The average age in Group A (TAP block) was 68.09 ± 9.47 years, in Group B (QL block) 67.69 ± 7.92 years, and in Group C (no regional block) 66.64 ± 7.37 years.

Similarly, the duration of surgery did not differ significantly between groups (*P* = 0.199), with mean operative times of 244.44 ± 58.01 minutes for Group A, 234.03 ± 55.14 minutes for Group B, and 248.61 ± 52.01 minutes for Group C. These findings reinforce the procedural standardization within the cohort, attributable to the single-center, single-surgical-team design.

In contrast, statistically significant differences were identified in estimated blood loss (*P* < 0.001), warranting further consideration. Patients in Group B (QL block) exhibited the lowest mean blood loss at 106.29 ± 69.99 mL, which was significantly lower than in both Group A (272.59 ± 296.54 mL) and Group C (239.31 ± 84.07 mL).

**Figure 1:**
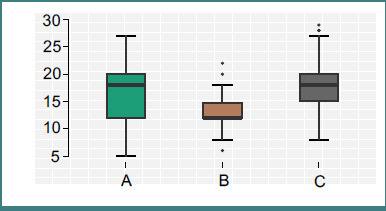
Opioid consumption. These differences underscore the opioid-sparing effect of the QL block. The narrower standard deviation in Group B also suggests greater consistency in analgesic efficacy, as compared to the broader variability observed in Group A. Notably, the reduction in opioid demand in Group B was statistically significant in post hoc comparisons versus both Group A (*P* < 0.001) and Group C (*P* < 0.001).

Total opioid consumption varied significantly (*P* < 0.001). Group B exhibited the lowest mean opioid dose (13.16 ± 2.69 mg), significantly less than Group A (16.80 ± 5.51 mg) and Group C (18.03 ± 4.29 mg) (Figure 1). The QL block, therefore, conferred a substantial opioid-sparing effect.

Group B patients demonstrated a prolonged analgesia-free interval, with a mean time to first analgesic dose of 16.06 ± 2.53 hours, compared to 8.11 ± 1.09 hours (Group A) and 5.50 ± 1.42 hours (Group C), *P* < 0.001.

The mean time interval from the end of surgery to the first administration of supplemental analgesia—used here as a surrogate marker of block efficacy and duration—significantly differed across study groups (*P* < 0.001).

**Figure 2:**
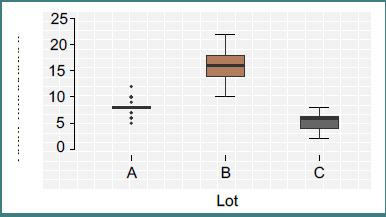
Time to first analgesic. Group B patients demonstrated a prolonged analgesia-free interval, with a mean time to first analgesic dose of 16.06 ± 2.53 hours, compared to 8.11 ± 1.09 hours (Group A) and 5.50 ± 1.42 hours (Group C), *P* < 0.001.

Patients in Group B (QL block) exhibited a prolonged analgesia-free interval, with a mean of 16.06 ± 2.53 hours (Figure 2). This was significantly longer than Group A (TAP block), where the mean time to first analgesia was 8.11 ± 1.09 hours, and Group C (no regional block), which exhibited the shortest interval at 5.50 ± 1.42 hours. This notable extension of pain-free duration in Group B—nearly double that of Group A and threefold compared to Group C—underscores the superior pharmacodynamic profile of the QL block in terms of onset and duration. The findings suggest that the anterior QL block provides a more robust and longer-lasting blockade of afferent nociceptive transmission, potentially due to its capacity to reach the thoracolumbar fascia and influence somatic and visceral fibers.

A comprehensive analysis of non-opioid analgesic consumption across study groups revealed statistically significant differences in the administration of both paracetamol and nefopam, as determined by the Kruskal–Wallis H test (*P* < 0.001).

Patients in Group B (QL block) demonstrated the lowest median total paracetamol consumption, with a central value of 3 grams over the postoperative observation period. In contrast, patients in Group A (TAP block) and Group C (no regional block) each had a higher median consumption of 4 grams, indicating a more frequent need for first-line non-opioid analgesia in these cohorts. Although paracetamol is routinely administered as the initial step in the departmental analgesic algorithm, the reduced consumption in the QL group may reflect both superior baseline analgesia and a delayed onset of breakthrough pain.

Similarly, nefopam, a centrally acting non-opioid analgesic commonly employed as a second-line agent, showed significant intergroup variation. The median dose administered in Group B was 20 mg, compared to 60 mg in Group A and 40 mg in Group C. This gradation strongly suggests that patients receiving QL blocks required fewer pharmacological escalations beyond paracetamol, reinforcing the notion of a more durable and effective regional block in this cohort.

In terms of tramadol and Diclofenacum + Orphenandrinum citras usage—typically reserved for third-line rescue analgesia in cases of moderate to severe pain—Group B patients received significantly fewer high-dose administrations. The 200 mg dose of tramadol and 750 mg dose of Diclofenacum + Orphenandrinum citras were substantially more prevalent in Groups A and C, whereas such escalated dosing was rarely necessary in Group B (*P* < 0.001, chi-square test). These findings support the analgesic efficacy of the QL block and underscore its potential in mitigating cumulative exposure to centrally acting agents and combination analgesics.

Finally, the length of postoperative hospitalization was markedly different among groups. Group B exhibited the shortest mean hospital stay, averaging 4.87 ± 1.14 days, followed by Group C at 5.46 ± 1.61 days and Group A at 6.91 ± 2.40 days. The difference reached statistical significance (*P* < 0.001), suggesting a potential association between superior analgesia—afforded by the QL block—and accelerated recovery trajectories. Reduced pain intensity and analgesic requirements likely facilitated earlier mobilization, resumption of oral intake, and fulfillment of discharge criteria, aligning with the goals of Enhanced Recovery After Surgery (ERAS) protocols.

These findings support the QL block not only as an effective analgesic modality but also as a potential driver of improved functional outcomes and resource optimization in minimally invasive colorectal surgery.

### Length of hospital stay

Hospital stay duration varied significantly between groups (*P* < 0.001). Post-hoc analysis showed:

- Group A had the longest average hospitalization (6.91 days),
- Group B had the shortest (4.87 days),
- Group C had intermediate values (5.46 days).

This suggests that the QL block may have facilitated faster postoperative recovery. Single-center design restricts external generalizability. Nevertheless, these findings substantiate the QL block as a valuable adjunct in colorectal anesthesia. Future prospective randomized controlled trials are warranted to validate these results, assess long-term outcomes, and optimize block technique and dosage.

## DISCUSSION

The present retrospective study evaluated the impact of two regional anesthesia techniques — the TAP block and the QL block — on postoperative analgesic requirements, pain control, and recovery outcomes in patients undergoing laparoscopic colon surgery. Our findings demonstrate that the QL block significantly reduced opioid consumption, delayed the need for the first analgesic dose, and shortened the length of hospital stay when compared to both TAP block and standard postoperative analgesic protocols [18].

These results are consistent with emerging evidence highlighting the superiority of QL block over TAP block in abdominal surgery. Anatomically, the QL block enables a wider spread of local anesthetic into the thoracolumbar fascia and potentially the paravertebral space, providing not only somatic but also visceral analgesia [19,20]. This mechanism likely explains the improved pain control and reduced analgesic demand observed in the QL group, particularly in the early postoperative period [21,22].

One of the most significant findings in our study is the prolonged time until the first analgesic administration in the QL group — approximately 8 hours longer than TAP and over 10 hours longer than the control group. This delay suggests that the QL block offers more durable analgesia compared to the TAP block, which is typically limited to the anterolateral abdominal wall due to its more superficial spread [23,24]. Early postoperative pain is a strong predictor of opioid requirement and functional recovery; thus, the ability of the QL block to maintain adequate pain control in the immediate postoperative period may have contributed not only to reduced opioid consumption but also to earlier mobilization and improved clinical recovery [25].

In terms of total opioid and non-opioid analgesic consumption, patients in the QL group consistently required the lowest doses across all measured agents, including paracetamol, tramadol, and nefopam. This finding is of clinical relevance, as minimizing opioid use is a cornerstone of ERAS protocols, given the well-documented risks of opioid-related adverse effects such as postoperative nausea and vomiting (PONV), delayed bowel function, respiratory depression, and chronic opioid dependence [26].

Interestingly, while the QL block appeared to reduce pain scores and medication requirements, it also correlated with the shortest length of hospital stay. Although the retrospective design of this study precludes definitive causal inference, this observation aligns with the hypothesis that effective perioperative pain management can expedite functional recovery, reduce immobilization-related complications, and enhance discharge readiness [27]. It is plausible that the superior analgesia afforded by the QL block allowed for earlier mobilization and initiation of oral intake, both of which are key discharge criteria in colorectal surgery pathways [28].

The observed difference in intraoperative bleeding, significantly lower in the QL block group, is intriguing but may reflect baseline surgical or anesthetic factors rather than the analgesic technique per se. Since the block is typically administered post-induction, its direct influence on intraoperative hemodynamics would likely be limited. Nevertheless, this variable was controlled in our multivariate analysis, reinforcing the validity of the analgesia-related outcomes.

Current literature robustly supports the abdominal Quadratus Lumborum (aQL) block as an effective intervention for reducing patient pain scores and total opioid consumption following abdominal surgeries. The analgesic benefits of this regional anesthesia technique are well-documented, with numerous studies demonstrating significant reductions in postoperative pain levels, which in turn leads to decreased reliance on opioids for pain management. While many studies primarily focus on pain scores and opioid use as key outcomes, two recent randomized controlled trials (RCTs) have provided compelling evidence that the aQL block may also contribute to improved hospital efficiency by reducing the length of stay (LOS) for patients. These trials indicate that patients receiving the aQL block not only experience enhanced pain relief but also benefit from a shorter recovery period in the hospital than those who do not [29-31].

The present study has several limitations inherent to its retrospective design. First, the lack of randomization introduces potential selection bias, as the choice of analgesic strategy was at the discretion of the attending anesthesiologist and surgeon. Second, the study was conducted at a single institution, which may limit the generalizability of the findings to other surgical settings or patient populations.

Despite these limitations, our findings add to the growing body of evidence suggesting that the QL block offers meaningful advantages over TAP block and systemic analgesia alone in laparoscopic colorectal surgery. Future prospective, randomized controlled trials are warranted to validate these observations and to further explore the optimal dosing, timing, and anatomical approach of the QL block to maximize patient outcomes.

## CONCLUSION

In summary, this retrospective analysis suggests that the QL block provides superior postoperative analgesia compared to the TAP block and conventional analgesic regimens in patients undergoing laparoscopic colon surgery. The technique was associated with lower opioid consumption, longer analgesia-free intervals, and reduced hospital stays. These findings support the inclusion of QL block as an effective component of multimodal analgesia strategies in minimally invasive abdominal surgery.

## Data Availability

The raw data supporting the conclusions of this article will be made available by the authors upon request.

## Personal thanks

This study is part of the doctoral research of Dr. Mihaela Roxana Olita, PhD student at the Doctoral School of Medicine of IOSUD Carol Davila University of Medicine and Pharmacy in Bucharest, with Dana Rodica Tomescu, MD, Ph.D, as scientific coordinator.
