# Supplementary material for: Regional anesthesia in colorectal laparoscopy: a retrospective comparison of quadratus lumborum and TAP blocks
Source: J Med Life. 2025 Apr;18(4):285–91. doi: 10.25122/jml-2025-0067 (PMC12094314; doi:10.25122/jml-2025-0067)
Supplement: Supplementary file 1 [file JMedLife-18-285-s001.pdf]

I. Anesthesia induction consisted of intravenous propofol (1.5–2.0 mg/kg), fentanyl (5 μg/kg) and rocuronium (0.6 mg/kg). Maintenance involved sevoflurane in oxygen/air, supplemental fentanyl boluses and rocuronium top-ups (0.1–0.2 mg/kg/h). Depth of anesthesia was continuously monitored using Bispectral Index (BIS), maintained between 40 and 60.

II. Laparoscopic surgeries were performed by the same surgical team, ensuring procedural uniformity. Pneumoperitoneum was established at 10–13 mmHg via a four or five port technique, including a 1-cm camera port.

© 2025 by the authors. This article is an open access article distributed under the terms and conditions of the Creative Commons Attribution (CC BY 4.0) license.
